# Observations on Fractures and Their Treatment1Read before the Syracuse Academy of Medicine, February 6, 1894.

**Published:** 1894-03

**Authors:** Gregory Doyle

**Affiliations:** 70 West Genesee Street; Syracuse, N. Y.


### OBSERVATIONS ON FRACTURES AND THEIR TREATMENT.[1]

By GREGORY DOYLE, M. D., Syracuse, N. Y.

IN NO branch of surgery does experience prove a better friend than in the treatment of fractures. Many of us, in our early days, have issued forth from the threshold of our *Alma Mater* with bright new diplomas in our eager hands, and full confidence in our undoubted ability to repair all forms of broken-up humanity; a colic or a furuncle presented no more difficulties than the most serious fracture. However, after a few surprises in the way of disastrous results we were convinced of the importance of extreme caution, careful attention and the assiduous study of each individual case.

The young photographer, after having read up on his art, thinks it only necessary to touch the button and take his chances as to the results. After he has spoiled a number of plates and a host of chemicals, it will perhaps dawn upon him that some extended experience and practical knowledge would beget better results and prevent many mistakes and much waste of good material.

Some forms of fracture are difficult, and require most careful examination to ascertain the exact condition of the injured parts.

---

1. Read before the Syracuse Academy of Medicine, February 6, 1894.

A good knowledge of anatomy is absolutely essential, for the origin and attachment of muscles must be taken into consideration, as also the proximity of vessels and nerves to the injured parts.

Tentative manipulation should be carefully and diligently made until the exact character of the lesion is ascertained, for if any part of the diagnosis is left to chance or guesswork, there may be subsequent cause for regret. When examining a fracture, all unnecessary suffering should be spared the patient. It is not necessary to turn and twist the limb or grind the broken ends together to get crepitation, as the unnatural outline of the injured member with its abnormal flexibility will, in most cases, determine the seat and nature of the fracture. The greatest gentleness should be used, as the patient is supersensitive to pain, and rough handling may produce dangerous irritation to the muscles and nerves and even rupture adjacent blood-vessels.

I remember seeing, in a distant city, a number of physicians trying to differentiate between a dislocated hip and an intracapsular fracture of the femur, which to the practised eye is a very easy matter. The patient was lying on a couch, surrounded by a throng of solemn-faced wise-looking inquisitors; each in his turn took a tug and a twist out of the poor fellow's leg in his efforts to make a diagnosis. This unnecessary and severe handling could have been avoided had they recognized in the shortened limb and everted foot a plain case of intracapsular fracture.

If any doubt should exist as to the real nature of the fracture, anesthetics should be used, that a thorough and satisfactory examination may be made without causing suffering to the patient or injury to the surrounding parts. While the muscles are thus relaxed and the patient is oblivious to pain, the fracture should, of course, be reduced.

Fractures near the joints are at the present day often mistaken for sprains or wrenches, especially near the wrist or ankle. Before the days of Dr. Colles, of Dublin, what is now known as Colles' fractures were treated as sprains, and the deformities resulting were very great and as numerous as the cases thus treated. After the investigation of Colles, Smith and others, this form of fracture was treated properly by those who studied and followed their methods.

To diagnosticate a fracture near the wrist, it is only necessary to place the forearms parallel to one another with the palms in apposition and compare the outlines of each. It is unnecessary and worse than useless to look for diagnostic crepitation, and in some cases it would be impossible to get it.

Many patients after being injured imagine, if power is left them for voluntary motion of the extremities, that there can be no possibility of a fracture. This unfortunate error has been the additional cause of many deformed and crippled limbs. People should know and remember that the extremities do not depend on the bones for their motion. Whole sections of bone may be absent in an arm without destroying the power of the hand or its usefulness. I have seen cases where large portions of the bony structure of the arm, even including the elbow joint, were absent without seriously curtailing the use of the hand. Many similar cases are reported in the surgical history of our late war.

At a recent meeting of the Imperio-Royal Society of Vienna, Prof. Billroth showed a man thirty-four years old, who, in spite of the entire absence of the shaft of the humerus, was able to perform his duties as a coachman. At the age of five years he had been thrown down by a carriage which passed over his arm, and protracted suppuration followed. At the time the patient was shown, the humerus was found to have been replaced by a hard cord as large as the thumb, probably containing the blood-vessels and nerves of the arm and perhaps some of its muscles. There were no trophic disturbances of either the forearm or hand.

On a late occasion I treated a very severe Colles' fracture in which existed great deformity. I felt diffident in assuming the case, as the outlook for a good result was not encouraging. However, after careful treatment and assiduous attention, it proved one of the best results I had ever obtained; the member was restored to usefulness without deformity.

When I sought the patient to grant me pecuniary acknowledgment, she utterly refused to do so, as the neighbors told her the arm was not broken at all, since she had suffered no pain after the first dressing and could move her fingers during the whole time. If the fracture had not been properly reduced, she would have suffered continuous pain, and if her fingers had been fastened to a palmar splint, as in the old manner of treatment, she could not have moved them at will. She and her neighbors would probably then feel they were getting an equivalent for the fees requested.

To facilitate the diagnosis of a fracture, it is necessary to learn the cause and conditions under which the injury was sustained.

This knowledge will often prove an invaluable aid in diagnosis and treatment.

There are certain well-known rules for the treatment of fractures which cannot be wholly ignored, but may be modified according to the exigencies of the case.

To attain the maximum of success, the surgeon must be fortified with a thorough knowledge of his duties and a natural taste or genius for his art. He must be endowed with the natural gifts of invention and construction. Otherwise he will prove a dismal failure, and the results of his work will prove a plague to him and a source of misery to his patient. Persons who are not naturally gifted with a taste for music or art could not attempt to sing a song or paint a picture without incurring the ridicule of their neighbors, but might not inflict serious injury to any one. Not so with the man of clumsy hands and untutored brain, who "rushes in where angels fear to tread," in his attempt to do the work of the skilful surgeon. Ugly deformities, life-long misery and expensive lawsuits are often the fruits of such overweening self-confidence.

It is better that a broken bone be reduced or set as soon as practical after the injury, as it is easier to adjust the fragments before much swelling occurs. If, however, a surgeon cannot be immediately had, the friends should place the limb in an easy position and apply warm water dressings until the arrival of some one duly qualified to attend to the case.

An accident generally produces much excitement among the neighbors, and perhaps five or six will rush to different telephones and hastily summon as many doctors. The first one that arrives, whether surgically qualified or not, stakes out the claim, as the miners would say, and holds the ground against all comers, even the family physician. As the proper treatment of a fracture is to the patient a matter of a life-time, he or his friends should calmly and without fear of offending any one, select some person of well-known surgical ability, even if a few hours were necessary in which to find him, as the patient will not suffer as much by a short or even tedious delay as he would at the hands of an unskilful or inexperienced operator. Dr. Samuel D. Gross says:

There is no class of injuries which a practitioner approaches with more doubt and misgiving than fractures. or one which demands a greater amount of ready knowledge, self-reliance and consummate skill. If I were called upon to testify what branch of surgery I regarded as the most trying and difficult to practise successfully and creditably, I should unhesitatingly assert that it was that which relates to fractures. As to myself, I never treat a case of fracture, however simple, without a feeling of the deepest anxiety in regard to its ultimate issue. I certainly know none which requires a more thorough knowledge of topographical anatomy, a nicer sense of discrimination, a clearer judgment, a more enlarged experience or a greater share of vigilance and attention—in a word, which requires a higher combination of surgical tact and power.

Such impressive language from one of the most illustrious fathers of American surgery ought to dispel any cherished ideas of the simplicity and ease with which broken bones may be repaired.

After dressing a recent fracture, the patient should in a short time feel much relieved. If not, it is very evident that the fractured surfaces have not been placed in apposition, and the surgeon should not feel that his duty is properly done until the patient is made comparatively comfortable, even if he has to undo the dressings and make a thorough examination and a readjustment if necessary.

Advance in the mechanical treatment of fractures has kept pace with the improvement of the age. New appliances and methods have replaced the old, less restriction is placed upon the sufferer, he is not imprisoned in cumbersome splints or manacled down, as it were, to his bed for weeks and months with strict orders not to move limb or muscle. As a result of intelligent observation and practical experience, it is now found proper to allow a patient liberties with his broken limbs that would utterly shock sticklers for old-time methods. The pistol splint has been fired into oblivion and the saw-dust box is relegated to the darkness of the lumber-room to give place to the results of cumulative experience.

The common practice of applying cold water dressings to injured or fractured limbs cannot be too strongly deprecated, although sanctioned by many surgical authors. Cold applications may, while in actual contact, keep down the temperature, but on their removal an injurious reaction is very apt to occur. Without minutely entering into this interesting subject, I would say that a surgical experience of nearly thirty years has taught me that warm applications only should be used in the treatment of local inflammations.

It may seem paradoxical that heat will expel heat or prevent its occurrence, but when applied to an injured surface it will prevent inflammatory action or reduce it much more effectually than cold. The moist warm poultices of our grandmothers have relieved more suffering than the ice bag fad of the present day. The industrious housewife who has been up to her elbows all day in the hot suds of the wash tub will find when her task is done at night that her hands are cool, white and comfortable, while her husband, who has been cutting ice on the lake, will, after the evening meal, display in sharp contrast a pair of inflamed hands, swollen to the size of boxing gloves, red as lobsters, and painful as aristocratic gout.

Warm water applied to the surface of a bleeding stump is seen to contract the fibers of the exposed muscles, and render them pale, while it arrests the bleeding. The elastic reticulated coats of the small arteries no doubt undergo the same contraction and thereby lessen or entirely close the mouths of the vessels. Chilly applications may do for the amphibious toad or the cold-blooded porpoise, but never for the warm heart's blood of the human biped.

As to the outcome and treatment of fractures, much, of course, depends on the character of the injury and the physical condition of the patient. In simple fractures it is to be expected that proper treatment will produce good results. There are, however, forms and degrees of fracture in which it is impossible to restore the limb to its former shape and usefulness, but often much may be accomplished in apparently hopeless cases. In these days of aseptic surgery, many crushed and mangled limbs may be saved that were formerly doomed to amputation.

In this most difficult branch of the healing art, surgeons are often blamed for deformed or shortened limbs. The faultfinder does not perhaps consider the history and extent of the injury, or if he does he expects something bordering on the miraculous. In many cases of simple fracture, especially of the femur, it is impossible to prevent more or less shortening, due, no doubt, to absorption of bone.

Many medical men, I am sorry to say, are prone to flippantly criticise the work of their brother practitioners without even enquiring into the history of the case or the extent of the injury. When invited to examine fractures that have been treated by my neighbors, I make it an invariable rule to acquaint the doctor in question before expressing an opinion, that is, when any deformities exist, and often find on investigation that the result is a very good one when the extent of the injury is taken into consideration.

The responsible duties of our exacting and laborious profession are often ameliorated by the kind words and deeds of professional brethren who are qualified to pass judgment on the results of our work. If we perform our duty properly, no unjust criticism of false friend or open foe need cause any concern.

70 West Genesee Street.

